# Translating WHO theory on alcohol to practice—where are we in the policy spectrum?

**DOI:** 10.1093/eurpub/ckag135

**Published:** 2026-07-25

**Authors:** Avi Magid

**Affiliations:** Department of International Health, Maastricht University, Maastricht, The Netherlands; Association of Schools of Public Health in the European Region (ASPHER), Brussels, Belgium

In 2023, the World Health Organization (WHO) stated that ‘no safe amount of alcohol consumption for cancers and health can be established’, and that ‘Alcohol consumers should be objectively informed about the risks of cancer and other health conditions associated with alcohol consumption’ [1]. Following this risk assessment, some countries consider taking (moving) it to public health policy in terms of guidelines and recommendations. Furthermore, some European countries have already recommended a total avoidance of alcohol consumption [2]. The manner in which this risk assessment should be translated into policy is a matter of ongoing debate forming a policy spectrum that ranges from informed individual decision-making to a rigid collective-centred precautionary approach.

This viewpoint proposes a foundational policy step common to all jurisdictions: acknowledging the WHO evidence regarding the absence of a ‘safe’ threshold. It should be borne in mind that the risks associated with low-level alcohol consumption are not uniform across populations. A GBD 2020 systematic analysis on alcohol found that the level of consumption that minimizes health loss is close to zero for younger adults across all world regions, while small amounts may be associated with reduced overall risk among older adults in regions with a high cardiovascular disease burden. This heterogeneity argues for age- and context-specific guidance rather than a single population threshold [3]. From this baseline, it presents diverse perspectives along the policy spectrum and calls for an informed debate among public health professionals regarding our collective position on this continuum.

The foundational step for a unified European policy is the formal acknowledgement of the WHO statement: there is no safe threshold for alcohol consumption. As a group 1 carcinogen, alcohol is classified alongside tobacco in the highest risk category, maintaining a strong causal association with seven types of cancer, including oesophageal, liver, colorectal, and breast cancer [1]. A direct consequence of adopting this risk assessment is the cessation of ‘moderate drinking’ as a safe health recommendation. Public messaging can no longer endorse ‘balanced’ drinking, particularly given the significant inter-individual variability in genetic predisposition, which makes it impossible to determine a safe limit for any specific individual.

While acknowledging the biological risks of ethanol constitutes a necessary scientific baseline, it does not dictate singular policy outcome. Instead, the translation of this evidence into public health actions exists along a broad policy spectrum, shaped by varying interpretations of state responsibility, individual autonomy, and cultural context. Identifying our current position on this spectrum is essential for a productive debate. To facilitate this, the following sections delineate the two contrasting ideological poles of this continuum: the individual-centred informed choice model and the collective-centred precautionary approach.

The individual-centred informed choice model focuses on the transparent communication of alcohol-related risks to the public, without necessarily imposing further regulatory mandates, beyond perhaps a general recommendation for reduction. This approach prioritizes individual agency, allowing the public to make autonomous lifestyle decisions based on their understanding of the risk-benefit trade-off. Much like the personal assessment of risk involved in crossing the street, this model posits that consumption choices should remain a private responsibility. By respecting established cultural habits and avoiding perceived overreach, this approach may be more effective in maintaining public trust, particularly given that tailored, high-resolution interventions are not feasible. However, the feasibility of this model is highly context-dependent. In countries with high per-capita consumption and severe alcohol-related harm, where the attributable risk of cancer is high, relying solely on individual agency may prove insufficient. In such high-burden setting, the limitations of the informed choice model become apparent, necessitating more robust, systemic interventions that move further along the policy spectrum toward precautionary pole.

At the opposite end of the spectrum lies the precautionary approach [2], which advocates for a regulatory framework similar to the WHO Framework Convention on Tobacco Control (FCTC) [4]. The rationale is two-fold: Biologically, as a Group 1 carcinogen, ethanol’s risk profile is fundamentally comparable to tobacco. Socially, proponents argue that alcohol consumption, much like second-hand smoking imposes significant ‘passive’ harms on others, including alcohol-related violence, road traffic accidents, and Foetal Alcohol Spectrum Disorders. Adopting this approach requires aggressive interventions: substantial tax increase, strict age limits, and treating alcohol industry as a ‘pariah’ stakeholder, analogous to Big Tobacco. However, the primary challenge of this model is its cultural dissonance. In many European regions, such as Italy, Portugal or Spain, alcohol is considered a cornerstone of cultural identity and agricultural heritage. Imposing ‘tobacco-style’ denormalization strategy in these contexts risks severe public resistance and may erode public trust.

The resolution of this tension does not lie in a universal, one-size-fits-all mandate, but rather in a strategic calibration along the policy spectrum. Each nation must determine its own position based on its unique epidemiological burden, cultural heritage, and public readiness. To guide this process, the WHO’s SAFER initiative provides a robust, evidence-based framework that allows for such flexibility. By focusing on five high-impact strategic areas Strengthening restrictions on availability, Advancing drink-driving countermeasures, Facilitating access to screening and treatment, Enforcing advertising bans, and Raising prices through taxation—the SAFER framework offers a menu of interventions that can be scaled according to a country’s specific needs [5].

While the biological evidence of ‘no safe amount’ is now a settled foundational step, the political and ethical journey toward effective implementation has only just begun. We must avoid the trap of binary thinking—choosing between total prohibition or passive inaction. Instead, I call for a transparent debate among public health professionals: How can we utilize tools like the SAFER initiative to protect health while respecting the social contract? By embracing this complexity, we can ensure that our recommendations remain both scientifically sound and socially sustainable, preserving the most vital ingredient of public health: the public trust.

Conflict of interest: The author declares that there are no conflicts of interest.

## Data Availability

Not applicable. This viewpoint does not involve any data.

## References

[1] Anderson BO , Berdzuli N, Ilbawi A et al Health and cancer risks associated with low levels of alcohol consumption. Lancet Public Health 2023;8:e6–7. 10.1016/S2468-2667(22)00317-6 36603913 PMC9831798

[2] Shield K , Stranges S. Going too far: why universal recommendations for alcohol abstinence may not be beneficial for public health. Eur J Public Health 2025;35:199–200. 10.1093/eurpub/ckae192

[3] Bryazka D , Reitsma MB, Griswold MG et al Population-level risks of alcohol consumption by amount, geography, age, sex, and year: a systematic analysis for the Global Burden of Disease Study 2020. Lancet 2022;400:185–235. 10.1016/S0140-6736(22)00847-9 35843246 PMC9289789

[4] Paraje G , Flores Munoz M, Wu DC et al Reductions in smoking due to ratification of the Framework Convention for Tobacco Control in 171 countries. Nat Med 2024;30:683–9. 10.1038/s41591-024-02806-0 38321222 PMC10957467

[5] The SAFER technical package: five areas of intervention at national and subnational levels. Geneva: World Health Organization, 2019. https://www.who.int/publications-detail-redirect/the-safer-technical-package

